# Dependence of the Atrioventricular Conduction Time on the Conduction through the Atrioventricular Node and His–Purkinje System

**DOI:** 10.3390/jcm12041330

**Published:** 2023-02-07

**Authors:** Bartosz Żuchowski, Krzysztof Błaszyk, Jarosław Piskorski, Andrzej Wykrętowicz, Przemysław Guzik

**Affiliations:** 1 Department of Cardiology Intensive Therapy, Poznan University of Medical Sciences, Przybyszewskiego 49, 60-355 Poznan, Poland; 2 I Department of Cardiology, Poznan University of Medical Sciences, Dluga 1/2, 61-848 Poznan, Poland; 3 Institute of Physics, University of Zielona Gora, Z. Szafrana 4a, 65-516 Zielona Gora, Poland

**Keywords:** atrioventricular conduction, atrioventricular node, His bundle, His–Purkinje system, electrophysiological study

## Abstract

The electrical depolarization of the heart passes through various structures of the cardiac conduction system, which modify its conduction to different extents. In this study, we investigated the relationship between the atrioventricular conduction time (AV interval) and its contributors, the atrioventricular node (AVN) and the His–Purkinje system (HPS), as represented by the AH and HV intervals, respectively. We also compared sex differences in these intervals and their relations. Resting intracardiac tracings lasting 5 min were obtained from 64 patients (33 women) during an invasive electrophysiological study. The aforementioned intervals were measured for all consecutive beats. The mean AH interval was 85.9 ms, HV 43.7 ms, and AV 129.6 ms. Men had longer AH (80.0 vs. 65.9 ms), HV (38.4 vs. 35.3 ms), and AV intervals (124.7 vs. 108.5 ms) than women. The AV intervals were linearly correlated with AH intervals in all patients (r^2^ = 0.65). No significant correlation was found between AV and HV intervals in all patients (r^2^ = 0.05). There were no sex differences in these associations. Our results suggest that the atrioventricular conduction time depends mainly on the conduction through the AVN and less on the HPS. These relations are similar in both sexes, although men had longer conduction times through the AVN, HPS, and total atrioventricular conduction time.

## 1. Introduction

In humans, the electrical impulse that initiates the coordinated contraction of cardiomyocytes originates from the sinus node. This impulse then travels through the atria, atrioventricular node (AVN), His bundle, and Purkinje fibers to reach the ventricles. During an invasive electrophysiological study (EPS), the conduction through the AVN is measured by the atrium-to-His (AH) interval, while the conduction through the His–Purkinje system (HPS) is measured by the His-to-ventricles (HV) interval. The combined AH and HV intervals form the AV interval, which represents the total atrioventricular conduction time. 

Although the AVN is smaller in size, the conduction through it lasts longer and is more influenced by the autonomic system and external factors compared to the HPS, which transmits depolarization waves rapidly. This suggests that the AVN and HPS may have unequal impacts on the total atrioventricular conduction time. However, the contribution of the AH and HV intervals to AVN conduction has not yet been quantitatively measured.

Previous studies have shown that the anatomy of the heart and cardiac conduction system differ between men and women, but data on sex differences in atrioventricular conduction is limited and conflicting [1,2,3,4,5,6]. In previous studies, AH, HV, and AV intervals were measured for a single cardiac cycle or a few selected cardiac beats. Such methodology is affected by a random selection of an insufficient amount of cardiac beats that may not reflect the average effect of AVN and HPS on atrioventricular conduction. Increasing the number of measured intervals and performing the analysis in a larger dataset should limit the impact of randomness and individual variance. 

To address these limitations, we analyzed the dependence of AV intervals on AH and HV intervals in a larger sample of data obtained from patients undergoing a clinically indicated EPS. We also studied sex differences in the duration of AH, HV, and AV intervals and the correlations between AV and AH and AV and HV. To ensure statistical power and accuracy, we measured AH, HV, and AV intervals in all consecutive sinus beats in 5 min recordings.

## 2. Materials and Methods

### 2.1. Study Group

This study is a retrospective analysis of intracardiac tracings from 64 patients aged 18–70 years who underwent a clinically indicated EPS at two independent electrophysiology labs in Poland. The EPS protocols for each procedure were chosen for specific clinical conditions and performed at the discretion of the electrophysiologist [7]. Indications for the EPS included documented or suspected cardiac arrhythmias or syncope with a probable cardiogenic background. All patients provided informed consent for the EPS and, if necessary, ablation. The Bioethical Committee at Poznan University of Medical Sciences approved this study as a retrospective analysis and agreed to use the data for an anonymous analysis. 

For this investigation, we used clinical data from consecutive patients who were chosen based on the following inclusion criteria.

- Sinus rhythm at the time of signal acquisition;
- Age: 18–70 years;
- Successful ablation or exclusion of cardiac arrhythmias during the EPS;
- Available information on the withdrawal of drugs affecting atrioventricular conduction for a least five half-life times before the EPS.

In addition, the following exclusion criteria were used:

- Any bundle branch block;
- Heart failure with left ventricle ejection fraction below 40%;
- Premature, atrial, or ventricular contractions constituting more than 5% of the recorded beats;
- Presence of a permanent cardiac pacemaker;
- Periodic deterioration of electrophysiological signal quality;
- Atrial fibrillation during the study.

### 2.2. Electrophysiological Study

The electrophysiological catheters were introduced through femoral or jugular vein punctures. All electrophysiological measurements were based on recordings from a 4-pole catheter with a 2-mm interelectrode distance. The catheter was located in the atrioventricular node region which recorded the sharp, largest available, His bundle electrogram (HBE), and standard surface ECG. For this study, 5 min fragments of continuous recordings, that were taken during the waiting phase after a successful ablation or an EPS that did not reveal any arrhythmia, were extracted from the electrophysiological system (LabSystem Pro, BARD/Boston Scientific, Marlborough, MA, USA). During that time, the catheters remained stable, no stimulation was performed, and the patients were resting in the supine position and breathing spontaneously.

### 2.3. Analysis of Recordings

**Figure 1:**
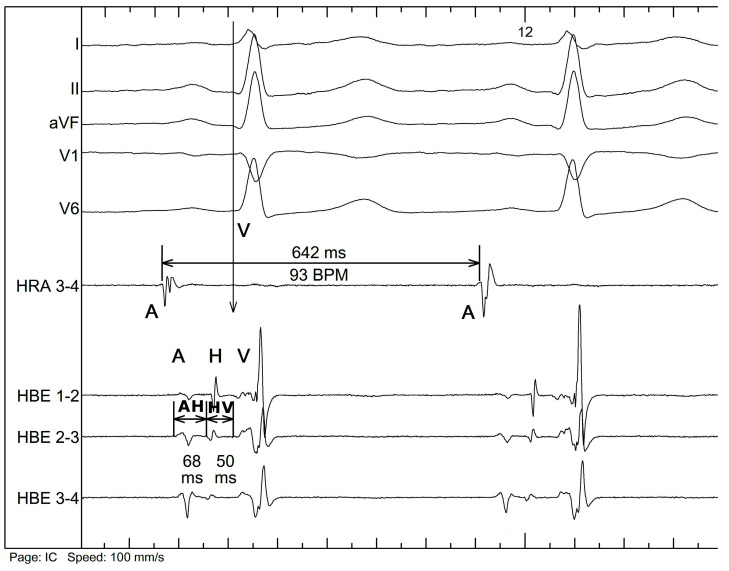
Example of signal labeling and interval measurement. HRA—high right atrium electrogram, HBE—His bundle electrogram, I, II, aVF, V1, and V6—surface ECG tracing. A: atrial signal, H: His signal, V: ventricular signal, AH: atrium-to-His interval, HV: His-to-ventricles interval (as explained in the text).

The extracted recordings were imported into LabChart Pro 7 (ADInstruments, Bella Vista, New South Wales, Australia) signal analysis software to perform measurements. The HBE catheter records three consecutive signals during each physiological cardiac cycle—A, H, and V. The A signal marks the moment when cardiac depolarization reaches the atrial input of AVN, the H signal marks the moment when cardiac depolarization exits the AVN and the depolarization of His bundle starts, and the V signal marks the moment when cardiac depolarization reaches the adjacent ventricular tissue after passing through the HPS. These signals, together with V from the surface ECG (corresponding to the earliest ventricular activation), allowed us to measure the following intervals (Figure 1) [7]:

- AH interval, measured from the A to H signal—corresponds to the conduction through the atrioventricular node.
- HV interval, measured from the H to earliest V signal—corresponds to the conduction through the His–Purkinje system.
- AV interval, measured from the A to earliest V signal—corresponds to the total atrioventricular conduction.
- AA interval, measured from the A to A signal of the consecutive cycle—corresponds to the interval between consecutive cardiac cycles, the equivalent of the heart rate.

All A, H, and V signals were marked in every beat of the 5 min recordings. Premature atrial, junctional, and ventricular beats were identified and affected intervals were excluded from further analysis. Depending on the heart rate, 254–656 measurements of AH, HV, and AV intervals were obtained from each patient.

### 2.4. Statistical Analysis

Data distribution was assessed using the Shapiro–Wilk test. Because the data were normally distributed, the mean values and standard deviations of each interval were calculated. The dependencies of AV intervals on AH and HV intervals were assessed using Pearson’s correlation and linear regression and compared using paired *t*-tests. Sex differences in conduction times and the relationships between AV and AH, and AV and HV were analyzed using *t*-tests for independent samples. The statistical analysis was performed using MedCalc version 19 (MedCalc, Ostend, Belgium). *p*-values of less than 0.05 were considered significant.

## 3. Results

### 3.1. Intracardiac Tracings

During the initial selection of recordings, multiple patients were excluded from the study due to failing to meet the restrictive inclusion criteria. From the initial 125 recordings, 31 were excluded due to temporary loss of A or H potential, 17 due to bundle branch block, and 13 due to exceeding the acceptable amount of premature atrial or ventricular beats. A total of 64 patients met all inclusion criteria and were included in the measurement of AH, HV, and AV intervals. Among them were 33 women and 31 men. On average, 383.7 measurements of each interval were performed per patient, resulting in a total of 24,558 measurements of each interval per patient and 73,674 measurements in total.

### 3.2. Study Group Characteristics

Table 1 summarizes the clinical characteristics of the studied patients. Among the 64 patients, no arrhythmia was induced during the EPS in 12 (18.7%) and, therefore, no ablation was performed. Atrioventricular nodal reentrant tachycardia was found and successfully ablated in 22 patients (34.4%), and Wolff–Parkinson–White syndrome was present in 22 patients (34.4%). Seven (10.9%) individuals were ablated due to premature ventricular contractions and one (1.5%) patient underwent ablation due to atrial tachycardia.

### 3.3. AH, HV, and AV Intervals Duration

Table 2 shows the summary of AH, HV, and AV intervals. The mean heart rate was 77 beats/minute, the mean AH interval was 86 ms, the mean HV interval was 44 ms, and the mean AV interval was 130 ms in the whole study group. Men had significantly longer minimal and mean AH, HV, AV, and maximal AH and AV intervals (Table 3).

### 3.4. Dependency of AV Intervals on AH and HV Intervals

**Figure 2:**
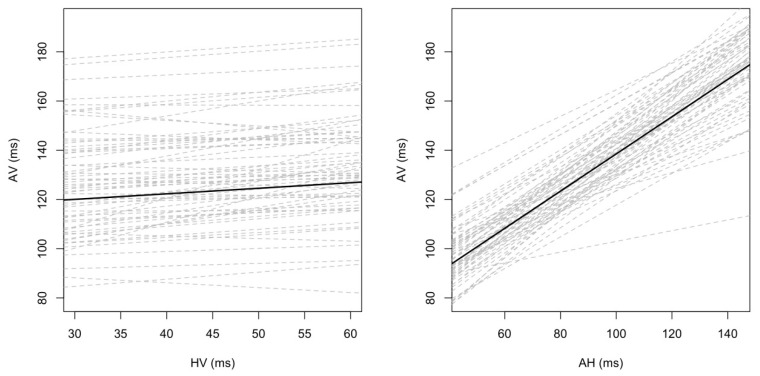
Dependency of AV intervals on AH and HV intervals. AH, HV, and AV—explained in the text.

AV intervals were significantly correlated only with AH intervals (*p* < 0.001). A direct comparison of the regression lines (r^2^—coefficient of determination) showed that the relations between AV and AH, and AV and HV differed significantly (*p* < 0.001) and that AV intervals depended more on the AH interval (r^2^ = 0.65; *p* < 0.001) than on HV interval (r^2^ = 0.05; *p* = 0.13) (Table 4, Figure 2). 

The analysis of AV~AH and AV~HV associations showed comparable results for men and women. In both sexes, a statistically significant correlation between AV and AH intervals was found (r^2^ = 0.65, *p* < 0.001) while the correlation between AV and HV intervals was not significant (r^2^ = 0.06; *p* = 0.23 for women, r^2^ = 0.04; *p* = 0.15 for men). The correlations between AV~AH and AV~HV differed significantly in each subgroup (*p* < 0.001) (Table 5).

A direct comparison of AV~AH and AV~HV dependencies between men and women did not reveal significant differences apart from the single intercept (b) value of linear regression (*p* = 0.01) (Table 6).

## 4. Discussion

### 4.1. The Dependency of Atrioventricular Conduction on the Conduction through the Atrioventricular Node and His–Purkinje System

We have observed a significant dominance of the atrioventricular node over the His–Purkinje system in modulating the total atrioventricular conduction time. Based on the r^2^ coefficient, 65% of the variance in the AV intervals is explained by the variance in the AH intervals, while only 5% is explained by the variance in the HV intervals. The ratio of r^2^ for the dependence of the AV interval on the AH interval and the HV interval is 13 to 1. Similar comparisons have not been studied before with the use of intracardiac recordings.

The AVN is a smaller structure than the long branches of His bundle and the dense net of Purkinje fibers. However, AVN appears to be a more potent modulator of the atrioventricular conduction time. The conduction through AVN is 50–60 times slower than through the HPS (0.02–0.05 m/s vs. 1–3 m/s). Anatomical differences in the structures of AVN and HPS are a probable explanation. The AVN’s structure is heterogenic, formed by multiple layers of cells. The proximal atrial part consists of 2–3 zones of transitional, or “fine”, cells connecting the atrial contractile cells and the cardiac conduction system [8]. These proximal, transitional cells are 2–4 times thinner than the contractile atrial cells [8]. Microscopic, non-conductive areas between cells might be responsible for the delay of conduction [9]. The middle part is the compact AVN lying on the fibrous cardiac skeleton. Distally, it continues into His bundle. The bundle of His consists of parallel cells surrounded by the fibrous tissue of the heart skeleton, forming an isolated track for fast depolarization wave conduction [10,11,12].

The gap junctions between the cardiomyocytes play an essential role in action potential propagation [13]. These are specialized membrane structures that provide chemical and electrical communication between adjacent cells [13]. Gap junctions are formed mainly by proteins called connexins [14]. Tracking the expression of connexin 43 (Cx43) in the atrioventricular junction has revealed the high complexity of signal conduction in this region [15]. The Cx43 expression in the compact AVN is lower than in the His bundle, resulting in fewer gap junctions and slower conduction [15]. Interestingly, the region known as the “slow pathway,” which is located in the inferior input to the AVN, has an increased expression of Cx43 [15]. This is probably because the fine cells in this area form a complex network with multiple end-to-end, side-to-side, and end-to-side connections, creating a labyrinth-like structure that delays the propagation of the depolarization wave despite having a high concentration of gap junctions [15]. 

The autonomic innervation also differs within AVN and HPS [16,17]. The sinus node has the best autonomic innervation and control of the cardiac conduction system. Alterations in the autonomic drive, i.e., from its parasympathetic and/or sympathetic contributors, modify the depolarization rate of the sinus node by lengthening or shortening the duration of the cardiac cycle. In contrast, atrioventricular conduction remains mainly under parasympathetic control. However, the distal structures of the conduction system are innervated, probably solely by sympathetic endings in humans, causing the vagal regulation of the HPS conduction to be marginal or none [17]. Tonkin et al. found that atropine shortens the AH interval by inhibiting the parasympathetic tone.

On the other hand, propranolol reduces the sympathetic influence on the heart and extends the AH interval duration by blocking the interaction of catecholamines with beta-adrenergic receptors [18]. Noteworthy, they noticed no significant effect of the atropine and propranolol on the HV interval. Alboni et al. also showed a discreet effect of the autonomic blockade on the AH interval, again with no effect on the HV interval [19]. Lately, it has been found that vagal denervation by cardioneuroablation shortens the AH intervals but does not affect the HV interval [20,21,22,23]. These findings might partially explain the uneven contribution of the conductions through the AVN and HPS to the total atrioventricular conduction.

### 4.2. Sex Differences in Atrioventricular Conduction Time

Men have longer conduction times through AVN, HPS, and total atrioventricular conduction time. Previous studies on this topic had conflicting results. In 2001, Liu et al. found that the AH, HV, and PR intervals were significantly longer in men than in women [2]. However, they used only five consecutive intervals from each patient in their measurements. Taneja et al. and Liuba et al. observed that the HV interval was longer in men, while AH did not differ between sexes [3,4]. Insulander et al. measured AH and HV intervals at rest, during mental stress, and during sympathetic inhibition by propranolol [5]. They found no sex differences in atrioventricular conduction at rest and during mental stress. However, after propranolol, the AH intervals were longer in men than in women. Suenari et al. studied 2088 patients undergoing ablation of atrioventricular nodal reentrant tachycardia and found that AH intervals were longer in men [6]. They also observed that younger women under the age of 50 had shorter AH intervals than older women.

Most studies do not provide information on how many cardiac cycles were used to measure each interval. It is, therefore, uncertain whether these studies accurately reflect the actual phenomena. In our study, we measured nearly 400 intervals per patient. Hundreds of measurements of each interval multiplied by 64 patients gave several thousands of AH, HV, and AV intervals. In this way, the effects of extreme values were limited, while average values better represented actual population values, including sex differences.

Different authors propose various possible mechanisms responsible for sex differences in AH and HV intervals. Women’s hearts are smaller than men’s, which translates to shorter conduction fibers and may be responsible for the shorter duration of these intervals. However, there is limited literature on this topic. Sex hormones, such as testosterone, progesterone, and estrogen, may also influence the ion channels and alter the electrophysiological features of the cardiac cells [6,24]. Cardiomyocytes have receptors for these hormones in their membranes, and some studies have shown that testosterone and progesterone shorten the action potential, while estrogen lengthens the QT interval [25,26,27]. More research is needed to understand the impact of sex hormones on cardiac conduction.

### 4.3. Sex Differences in the Relation of Atrioventricular Conduction to Conduction over the Atrioventricular Node and the His–Purkinje System

The only significant difference between men and women was found in the b-value (intercept) for the AV~HV correlation (*p* = 0.01). This difference may be due to the distinct y-axis intercept of the regression line caused by the difference in conduction times. Since the remaining coefficients did not differ, the influence of AVN and HPS on total atrioventricular conduction appears comparable in women and men. This issue has not been investigated previously.

### 4.4. Clinical Implications

The results of this study increase our understanding of the contribution of the different structures in the cardiac conduction system to atrioventricular conduction. We believe that knowledge of these relations might help predict the potential effects of modifying conduction (with pharmacotherapy or invasive ablation) in specific structures of the cardiac conduction system.

The effects of non-invasive (carotid sinus massage, Valsava maneuver) or invasive (electrophysiological ablation or cardioneuroablation) interventions on AV conduction can be quantitatively monitored with the EPS.

Current reference values for AH, HV, and AV intervals are the same for men and women. However, we show that the mean values differ significantly between sexes—even 15 ms for AH and AV intervals. Since the absolute values of the measured intervals are critical in interpreting EPS results, it is plausible that sex differences in AV conduction should be considered. If borderline or abnormal AV conduction is found, then pacemaker implantation may be unnecessary or premature in a man but demanded in a woman. So far, no one has proposed such an approach. However, such a conclusion is based only on the theoretical analysis of our findings. Before becoming a clinical practice, it should be prospectively tested in clinical studies.

We demonstrate quantitatively that the atrioventricular node and His bundle do not have equal inputs to AV conduction. Additionally, the described contributions to AV conduction are similar between men and women.

Precise measurement of AV conduction may aid in understanding how it is affected by various pathologies, such as ischemic heart disease, inflammation, amyloidosis, hypertrophic cardiomyopathy, or myocardial fibrosis. Intracardiac conduction abnormalities may complicate these clinical conditions.

Detailed AV conduction analysis can aid in understanding the effects of various pharmacological treatments and interventions on AV function. These include direct treatments, such as class I antiarrhythmics and amiodarone, and indirect treatments, such as cardiotoxic chemotherapeutics. Additionally, non-invasive interventions, such as carotid sinus massage and Valsava maneuver, and invasive interventions, such as electrophysiological ablation or cardioneuroablation, can also modify AV function. However, whether these treatments and interventions have a similar impact on the AV node and the His–Purkinje system is uncertain.

### 4.5. Study Limitations and Future Directions

Intracardiac tracings were obtained from patients shortly after an invasive electrophysiological study or ablation, which can be associated with emotional stress and pain. Thus, patients were given an anxiolytic (a benzodiazepine) prior to the procedure. In cases of ablation, they were given an analgesic (fentanyl). These circumstances are not entirely physiological and might affect the measured intervals. However, performing a similar study on healthy people only for scientific purposes would be unethical; thus, our approach is the next best option.

Some measurements were obtained from patients who underwent “slow pathway” ablation or modification due to AVNRT. Ablation in this region can potentially modify the signal conduction in the atrioventricular node. To prevent distortion of the result, during early recordings, we calculated the mean values of five consecutive AH, HV, and AV intervals before and after the ablation in these patients. Any intracardiac tracings with a difference of over 20% were excluded from the analysis.

All measurements were performed manually, which can be challenging due to catheter movement during heart contractions and the complex morphology of each signal. We excluded recordings with unstable signals that significantly changed between beats to increase precision. Many studies have confirmed that manual measurements are reliable [28].

Our study was conducted on patients who discontinued medications affecting the AV node. However, anxiolytics and/or analgesics given to patients might have affected the results. Therefore, the true and unmodified impact of the autonomic system on AV conduction is still uncertain. A similar study on patients under pharmacological autonomic blockade might provide new information and insight into AV conduction. Additionally, studying AV conduction during cardioneuroablation may also be of interest.

It is now possible to study AV conduction in real-life conditions. The most advanced cardiac implantable electronic devices can now constantly monitor AH and HV activities, providing a unique opportunity to study AV conduction physiology in a natural patient environment, both day and night, without any medications. These are just some examples of the potential implications of our findings.

The His potential recording was obtained with a standard quadripolar catheter with a 2-mm interelectrode distance. Care was taken to record the earliest His potential. Novel multipolar catheters with very small electrodes dedicated for ultra-high density mapping may provide even more precision in His bundle recording. However, such modern catheters are not commonly used in EPS.

Interesting results may be obtained by matching patients based on age, sex, body size by height or BMI, and physical activity. Our retrospective investigation used data from consecutive individuals as its principal assumption, reducing potential biases from patient preselection and matching. Subdividing them into smaller age- and BMI-matched groups might significantly reduce the statistical power. Further exploration of the effects of age, body size, and physical activity on AV conduction is of great clinical significance and should be a focus of future research.

## 5. Conclusions

Atrioventricular conduction time is largely determined by conduction through the atrioventricular node. On average, 65% of the variance in atrioventricular conduction time is explained by the variance in atrioventricular node conduction time. Only 5% of the variance of AV conduction is explained by the His–Purkinje conduction time. Although these correlations do not differ significantly between the sexes, conduction times through the atrioventricular node and the His–Purkinje system are longer in men than in women. Sex differences should probably be considered when interpreting borderline EPS results and determining the need for pacemaker implantation. Further clinical studies are needed to confirm these conclusions.

## Figures and Tables

**Table 1:** Clinical characteristics of the studied patients.

	Mean	SD	Min	Max
Age, years	39.8	15.3	18	70
Body mass, kg	74.3	14.7	50	104
Height, cm	171	8.5	156	194
BMI, kg/m^2^	25.3	4.0	17.3	33.7

Abbreviations: kg—kilograms, cm—centimeters, m^2^—square meters, SD—standard deviation, Min—minimal value, Max—maximal value. BMI—body mass index.

**Table 2:** Summary of the duration of AH, HV, AV, and AA intervals in the whole study group.

Interval	Mean [SD]
Minimum values	
AH, ms	72.7 [17.3]
HV, ms	36.8 [5.6]
AV, ms	116.4 [19.7]
AA, ms	617.5 [133.2]
Mean values	
AH, ms	85.9 [18.3]
HV, ms	43.7 [6.4]
AV, ms	129.6 [21.2]
AA, ms	780.5 [140.1]
Maximum values	
AH, ms	99.4 [20.5]
HV, ms	51.3 [6.7]
AV, ms	141.9 [23.0]
AA, ms	910.7 [184.2]

Abbreviations: ms—milliseconds, SD—standard deviation. AH, HV, AV, and AA—explained in the text.

**Table 3:** Comparison of AH, HV, AV, and AA interval duration between men and women.

Interval	WOMEN	MEN	
	Mean [SD]	Mean [SD]	*p*-Value
Minimum values			
AH, ms	65.9 [14.0]	80.0 [17.9]	<0.001
HV, ms	35.3 [5.4]	38.4 [5.6]	0.03
AV, ms	108.5 [15.8]	124.7 [20.4]	<0.001
AA, ms	597.4 [127.6]	639.0 [137.8]	0.22
Mean values			
AH, ms	79.0 [14.7]	93.2 [19.1]	0.001
HV, ms	42.2 [5.8]	45.3 [6.7]	0.049
AV, ms	121.3 [16.1]	138.5 [22.4]	<0.001
AA, ms	754.1 [130.3]	808.7 [146.6]	0.12
Maximum values			
AH, ms	92.6 [17.6]	106.6 [21.1]	0.01
HV, ms	50.2 [6.5]	52.4 [6.8]	0.19
AV, ms	133.9 [18.9]	150.4 [24.2]	0.01
AA, ms	873.7 [168.0]	950.2 [195.0]	0.10

Abbreviations: ms—milliseconds, SD—standard deviation. AH, HV, AV, and AA—explained in the text.

**Table 4:** Comparison of dependencies of the AV interval on AH and HV intervals for the whole study group.

	AV on AH	AV on HV	
Value	Mean	Mean	*p*-value
a	0.72	0.24	<0.001
b	63.70	111.70	<0.001
r^2^	0.65	0.05	<0.001
*p*	<0.001	0.13	

Abbreviations: a—slope, b—intercept, r^2^—coefficient of determination. AH, HV, and AV—explained in the text.

**Table 5:** Comparison of differences in the regression lines for AV~AH and AV~HV in men and women.

**WOMEN**			
	AV on AH	AV on HV	
	Mean	Mean	*p*-value
a	0.72	0.23	<0.001
b	63.70	111.70	<0.001
r^2^	0.65	0.06	<0.001
**MEN**			
	AV on AH	AV on HV	
	Mean	Mean	*p*-value
a	0.71	0.24	<0.001
b	71.10	127.3	<0.001
r^2^	0.65	0.04	<0.001

Abbreviations: see Table 4.

**Table 6:** Comparison of AV~AH and AV~HV dependencies between men and women.

	WOMEN	MEN	
	Mean	Mean	*p*-Value
AV on AH			
a	0.72	0.71	0.94
b	63.70	71.10	0.06
r^2^	0.65	0.65	0.95
AV on HV			
a	0.23	0.24	0.81
b	111.70	127.30	0.01
r^2^	0.06	0.04	0.50

Abbreviations: see Table 4.

## Data Availability

Not applicable.
